# Neck circumference may be a valuable tool for screening individuals with obesity: findings from a young Chinese population and a meta-analysis

**DOI:** 10.1186/s12889-018-5448-z

**Published:** 2018-04-20

**Authors:** Xiaoting Pei, Li Liu, Mustapha Umar Imam, Ming Lu, Yanzi Chen, Panpan Sun, Yaxin Guo, Yiping Xu, Zhiguang Ping, Xiaoli Fu

**Affiliations:** 1 College of Public Health, Zhengzhou University, No. 100 of Science Avenue, Zhengzhou, 450001 Henan China; 2 School of Basic Medical Sciences, Zhengzhou University, Zhengzhou, 450001 China; 3 Department of Medical Biochemistry, Faculty of Basic Medical Sciences, College of Health Sciences, Usmanu Danfodio University, Sokoto, Nigeria; 4 Nursing Department of Jiaozuo People’s Hospital, Jiaozuo, 454150 China

**Keywords:** Neck circumference, Central obesity, Overweight/obesity, Receiver operator characteristic curve

## Abstract

**Background:**

Central obesity and overweight/obesity can result in various chronic non-communicable diseases, such as cardiovascular disease, metabolic syndrome, and diabetes mellitus. Waist circumference (WC) and body mass index (BMI) are widely used to measure obesity despite their limitations. For example, WC and BMI cannot be measured in pregnant women and subjects with abdominal ascites or masses. Therefore, this study aims to determine the efficacy of neck circumference (NC) as a tool for screening central obesity and overweight/obesity.

**Methods:**

A total of 1169 undergraduates aged 18–25 years were studied by a cross-sectional survey in China, 2016. Questionnaires and physical examinations were used to collect data. Receiver operator characteristic (ROC) curve was performed to determine the best threshold of NC for screening central obesity and overweight/obesity. Meanwhile, a meta-analysis was conducted to estimate the efficacy of NC for screening central obesity and overweight/obesity synthetically.

**Results:**

NC was moderately correlated with WC and BMI. The ROC analysis showed that 37.1 cm for male and 32.6 cm for female were the best thresholds for central obesity, and 37.4 cm and 32.2 cm for overweight/obesity, respectively. The sensitivity, specificity, area under receiver operating curve (AUC) of central obesity and overweight/obesity were higher. In the meta-analysis, the pooled sensitivity, specificity, AUC and their *95%CI* of NC for screening central obesity were 0.72 (0.68~ 0.75), 0.87 (0.74~ 0.94), 0.77 (0.73~ 0.80) for male and 0.73 (0.65~ 0.80), 0.80 (0.71~ 0.86), 0.82 (0.79~ 0.86) for female. For overweight/obesity, the pooled sensitivity, specificity, AUC and corresponding *95%CI* were 0.83 (0.70~ 0.91), 0.77 (0.66~ 0.85), 0.86 (0.83~ 0.89) for male and 0.82 (0.71~ 0.90), 0.84 (0.61~ 0.95), 0.89 (0.86~ 0.92) for female.

**Conclusion:**

NC may not be a good tool for screening individuals with central obesity. But it may be a simple and valuable tool for screening individuals with overweight/obesity, especially in females.

## Background

Central obesity is a medical condition in which excess abdominal fat accumulates resulting in increased waist size, while overweight/obesity occurs when weight is higher than what is considered healthy weight for a given height (http://www.cdc.gov/obesity/adult/defining.html). Both of these conditions can result in various chronic non-communicable diseases (CNCDs), such as cardiovascular disease (CVD), metabolic syndrome (MS), and diabetes mellitus (DM) [1–4]. Obesity has become a worldwide and major public health problem due to increasing prevalence. Data from World Health Organization (WHO) showed that in 2016, more than 1.9 billion adults were overweight [5]. In China, the prevalence of obesity was 10.1% for Chinese urban adults in 2015 [6]. Compared with hypertensive patients with normal weight, the probability of suffering from hypertension complications in patients with obesity and overweight is increased by 11.65% and 6.45%, respectively. The per capita annual medical expenses due to overweight and obesity are 1410 and 985 RMB respectively, accounting for 22.79% of the medical expenses of hypertension complications [7]. For diabetes, the economic burden caused by diseases attributable to systemic obesity and central obesity are 11.2 and 38.8 million, respectively [8].

Fortunately, people with obesity can be treated appropriately, thereby reducing the incidence of CNCDs and greatly improving the living quality of individuals. Therefore, it is necessary and helpful to screen individuals with obesity at early stages. Waist circumference (WC) is widely used to measure central obesity while body mass index (BMI) is used as an indicator of overweight/obesity [9]. There are sustained efforts by researchers to find better indices for screening obese subjects due to the limitations of WC and BMI [10, 11]. For example, the diagnosis of central obesity cannot be made on pregnant women and subjects with abdominal ascites or masses using WC, which can also be influenced by many other factors including meal, respiration, or health conditions. Furthermore, BMI cannot determine body fat distribution nor can it be used to distinguish between muscle and body fat mass, which is the reason why athletes tend to have higher BMI readings suggestive of overweight/obesity, even though their extra weights are due to increased muscle mass not fat.

Neck circumference (NC) is recognized as a screening measure for identifying obese individuals [12–14]. In fact, recent studies have focused on its diagnostic accuracy for central obesity and overweight/obesity [15–17]. However, the results of these studies have been inconsistent possibly due to differences in the study population, diagnostic criteria and lifestyle. Additionally, the sensitivity, specificity, diagnostic odds ratios (DORs) and the area under the receiver operating curve (AUC) of NC as a measure of central obesity and overweight/obesity have not been reported in a meta-analysis. Thus, this study evaluated the efficacy of NC as a measure of central obesity and overweight/obesity through a cross-sectional survey that was analyzed with other similar studies to form a meta-analysis using a hierarchical summary receiver operator characteristic (HSROC) model. This study was designed to explore a universally applicable and convenient method of screening central obesity and overweight/obesity, so that appropriate measures can be taken on time to slow down the progression of obesity and related disease. It is hoped that the findings can have huge implications on prevention of CNCDs.

## Methods

### Sample introduction

A two stage cluster sampling cross-sectional survey was carried out in Zhengzhou University, China, 2016. In the first stage, 11 out of 47 departments were selected by stratified sampling, while in the second stage, three classes were selected from each department by cluster sampling. Undergraduates aged from 18 to 25 years old who participated in the study were asked to fill up a questionnaire and had physical examinations. Calculated sample size by PASS based on cross-sectional study was 914, in which, the prevalence of obesity was defined as 10% [18], the significance level and allowable error were set at 0.05 and 0.01, respectively, (the calculated sample size based on ROC analysis was 420). A margin of 20% was given for the sample size to cover for any invalid questionnaires. In total, 1207 questionnaires were returned out of 1215 questionnaires that were sent out. Individuals with goiter or other neck masses and deformity were excluded. The questionnaires without any of the following variable values were regarded as invalid ones: gender (*n* = 0), age (*n* = 0), height (*n* = 26), weight (*n* = 26), neck circumference (*n* = 38) and waist circumference (*n* = 37), which were excluded from further analysis. Finally, 1169 questionnaires were included in the final analysis. The response rate was 96.2%. The protocol for the study was approved by the ethics committee of Zhengzhou University, and written consents were obtained from all the participants.

### Data collection

Each participant was required to fill up a questionnaire providing detailed demographic characteristics, lifestyle risk factors and other information, while anthropometric data was measured by trained staff according to the unified standards [19, 20]. Height and weight were measured with light clothing and without shoes, after emptying of bladder. The WC was measured on standing participants with light clothing at the level of 1.0 cm above the navel while the hip circumference (HC) was taken at the maximal level of the hip. NC was measured by applying a tape around the inferior margin of the thyroid cartilage and perpendicular to the long axis of the neck with the participants standing, head erect and eyes looking forward horizontally [10]. The body fat percentage (BFP) was determined using the handheld Omron Body Fat Analyzer HBF-306. Height, WC, HC, NC were measured to the nearest 0.1 cm. Weight was measured to the nearest 0.1 kg and BFP was measured to the nearest 0.1%. BMI was calculated as weight (kg) divided by the square of height (m^2^), and waist-to-hip ratio (WHR) was also calculated. Each anthropometric indicator was measured three times by the same staff. The average of the three measurements was regarded as the value of each one.

### Definitions of central obesity, overweight/obesity and covariate factors

Central obesity was defined as WC ≥ 85 cm in males and ≥ 80 cm in females [21]. Overweight was defined as 24 kg/m^2^ ≤ BMI < 28 kg/m^2^ and obesity was defined as BMI ≥28 kg/m^2^ [16]. Current smoking was defined as having smoked 100 cigarettes and smoking cigarettes currently. Drinker was classified into no history of drinking, occasionally drinker (1–3 drinking days/week) and frequent drinker (4–7 drinking days/week) [22].

### Data extraction for meta-analysis

A meta-analysis was performed according to the guidelines of the Preferred Reporting Items for Systematic reviews and Meta-Analyses (PRISMA) [23]. Relevant studies in the Web of Science, PubMed, PubMed Central, China National Knowledge Infrastructure (CNKI) (Chinese) and Wanfang (Chinese) databases were searched up to October 31st, 2017 (date of publication) by using the search terms ‘neck circumference’, ‘obesity’, ‘receiver operating curve’, ‘waist circumference’, ‘body mass index’ and these terms’ derivation and combinations. The following inclusion criteria were used: (1) cross-sectional study, case-control study, cohort study, or diagnostic test that has evaluated the diagnostic value of NC in central obesity or overweight/obesity and gave the diagnostic threshold for central obesity and overweight/obesity; (2) the participants of the study were adults (≥18 years old); (3) the number of total sample, true positive value (TP), false positive value (FP), true negative value (TN) and false negative value (FN) can be obtained directly or indirectly. (4) ROC analysis was stratified by gender. Exclusion criteria were: (1) diagnostic criteria was unclear; (2) reviews, editorials, commentaries, or reports etc.; (3) duplicates within and between the databases, that is, repeating data that were already reported by other included articles. For all selected studies, relevant information including the first author, year of publication, study location, year of survey, the effective sample size, age range, diagnostic criteria, TP, FP, TN, and FN (male/female) was extracted. When the data could not be extrapolated, the corresponding author was contacted by e-mail.

### Statistical analysis

Statistical analyses were conducted using Stata, V12.0 (Stata Corp, College Station, Texas, USA). Chi-squared test or Student’s unpaired *t*-test was performed to determine differences between two groups. Receiver operating characteristic (ROC) curve was used to evaluate the performance of NC in identifying individuals with central obesity and overweight/obesity. Youden’s Index [24] was used to determine the best cut-off points for NC screening of individuals with central obesity and overweight/obesity. All *P*-values were 2-tailed, and the significance level was set at *α* = 0.05. The meta-analysis was performed to estimate the pooled sensitivity, specificity, DOR and AUC, and their corresponding 95% confidence intervals (CIs) based on the random effect model with HSROC. For this model, the total variation was partitioned into within- and between-studies components. Each component contained a systematic part and random part. HSROC appropriately weighed studies to account for within-study variability and used a random-effects approach to account for between-study variability. In addition, the summary curves were adjusted for covariates [25, 26]. All of these were different from the traditional model, SROC. To explore potential publication bias, the Egger’s tests were conducted.

## Results

### Characteristics of the participants

The 1169 participants consisted of 641 males and 528 females. Of these, 116 (9.9%) individuals were central obesity. Also, 177 (15.1%) individuals were with overweight/obesity. There were statistical differences between males and females in central obesity, overweight/obesity, smoking and drinking status (*P* ≤ 0.001) (Table 1). The BMI, NC and WC in males were significantly higher than those in female (*P* < 0.001).

**Table 1:** Comparison of demographic characteristics between male and female [*n*(%)]

Variable		Male (*n* = 641)	Female (*n* = 528)	*χ* ^*2*^	*P*
Central obesity	No	555 (86.6)	498 (94.3)	19.378	< 0.001
	Yes	86 (13.4)	30 (5.7)		
Overweight/ obesity	No	524 (81.7)	468 (88.6)	10.694	0.001
	Yes	117 (18.3)	60 (11.4)		
Smoking	No	548 (85.5)	520 (98.5)	61.924	< 0.001
	Yes	93 (14.5)	8 (1.5)		
Drinking	Never	109 (17.0)	307 (58.1)	218.069	< 0.001
	Occasionally	485 (75.7)	212 (40.2)		
	Frequently	47(7.3)	9 (1.7)		

### Correlation analyses between NC and anthropometric measurements

For all participants, Pearson correlation coefficients were calculated between NC and other indicators related to obesity, including WC, BMI, BFP and WHR. It showed that NC correlated positively with all of these indicators (*P* < 0.001). After stratifying the participants by gender, we also found a positive correlation between NC and obesity indicators (WC, BMI, BFP and WHR) (*P* < 0.001). The correlation coefficients between NC and WC were maximal in both male and total participants (*r* = 0.626 in male and *r* = 0.721 in total). The results of correlation analyses are shown in Table 2.

**Table 2:** Correlation coefficients between neck circumference and other indicators related to obesity

Parameters	Male (*n* = 641)	Female (*n* = 528)	Total (*n* = 1169)
WC^*^	0.626	0.604	0.721
BMI^†^	0.600	0.635	0.505
BFP^‡^	0.142	0.347	0.258
WHR^§^	0.368	0.300	0.630

*WC* waist circumference, *BMI* body mass index, *BFP* body fat percentage, *WHR* waist-to-hip ratio

All *P* values of correlation coefficients were less than 0.05

### ROC analysis and optimal cut-off points of NC for central obesity and overweight/obesity

**Fig. 1:**
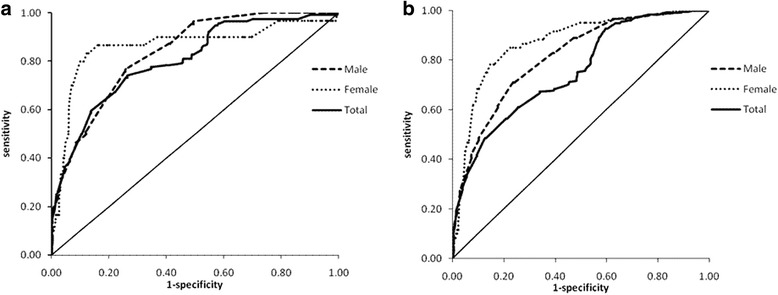
ROC curves for NC screening central obesity and overweight/obesity ((**a**) ROC curves for NC screening central obesity, the sensitivity, specificity and optimal cut-off point were 0.767, 0.741, 37.1 cm and 0.833, 0.878, 32.6 cm for male and female respectively. For total, the sensitivity, specificity and optimal cut-off point were 0.741, 0.735 and 36.1 cm. **b** ROC curves for NC screening overweight/obesity, the sensitivity, specificity and optimal cut-off point were 0.709, 0.763, 37.4 cm and 0.783, 0.853, 32.2 cm for male and female respectively. For total, the sensitivity, specificity and optimal cut-off point were 0.486, 0.873 and 37.5 cm)

The ROC analysis was used to determine the optimal cut-off points of NC for diagnosing central obesity and overweight/obesity. According to the Youden’s Index, NC ≥ 37.1 cm for male and ≥ 32.6 cm for female were determined to be the best cut-off points for screening individuals with central obesity, with a sensitivity of 0.767, and a specificity of 0.741 for male, and 0.833 and 0.878 for female, respectively. AUC and corresponding *95%CI* were 0.835 (0.795~ 0.875), and 0.863 (0.775~ 0.950) for male and female, respectively. The positive likelihood ratio (+LR) and DOR were 2.96, and 12.49 in male, and 6.83 and 35.82 in female, respectively. The ROC curves related to NC and central obesity for male and female are presented in Fig. 1. For overweight/obesity, the ideal cut-off points of NC were 37.4 cm for male and 32.2 cm for female, with a sensitivity of 0.709, and a specificity of 0.763 for male, and 0.783 and 0.853 for female, respectively. AUC and corresponding *95%CI* were 0.811 (0.770~ 0.852), 0.871 (0.823~ 0.918) for male and female, respectively. The +LR and DOR were 2.99, and 7.87 in the male group and 5.33, and 8.61 in the female group, respectively. The ROC curves related to NC and overweight/obesity for male and female are presented in Fig. 1.

### Meta-analysis

**Fig. 2:**
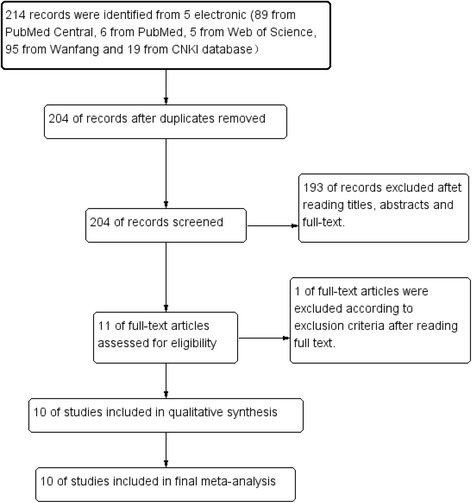
The flow diagram of the study identification and selection

We identified a total of 214 records from 5 electronic databases according to our search strategy (89 from PubMed Central, 6 from PubMed, 5 from Web of Science, 95 from Wanfang and 19 from CNKI database). Then, 10 duplicates were removed from the initial records, and another 193 irrelevant records were removed through primary screening of titles, abstracts or full text. Eleven articles were left for data extraction and further evaluation. One out of the 11 publications was removed according to exclusion criteria after reading the full text. Finally, 10 publications with 26 studies were included in the meta-analysis, because there were two or more subgroup analyses in some publications. The flow diagram of the study identification and selection is presented in Fig. 2.

**Fig. 3:**
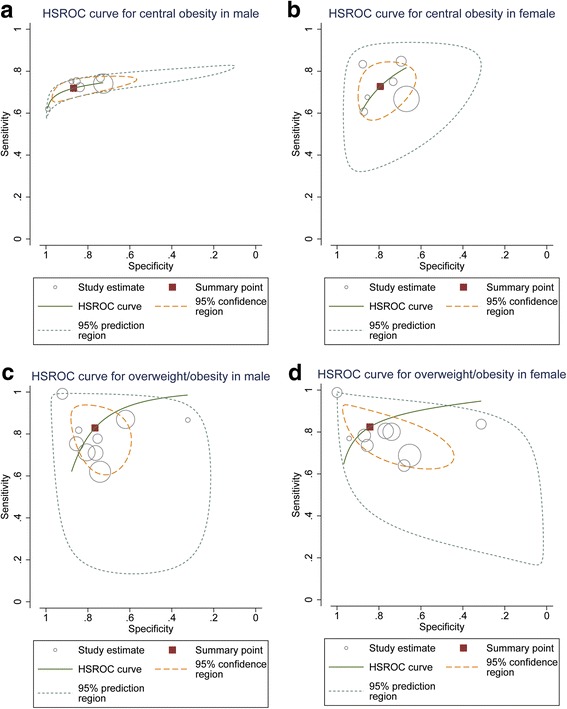
The HSROC curves of NC for screening central obesity and overweight/obesity ((**a**) the HSROC curve of NC for screening central obesity in male; (**b**) the HSROC curve of NC for screening central obesity in female; (**c**) the HSROC curve of NC for screening overweight/obesity in male; (**d**) the HSROC curve of NC for screening overweight/obesity in female)

Five [27–31] and eight [16, 29–35] publications were selected for central obesity and overweight/obesity, respectively. In order to increase the sample size and representativeness, we incorporated our own research into this meta-analysis. A total of six and nine articles were included for meta-analysis. Detailed characteristics of included studies are summarized in Table 3. For central obesity, 14,956 participants were included in the meta-analysis, out of which 8417 individuals had central obesity. The pooled sensitivity, specificity and AUC (*95%CI*) were 0.72 (0.68~ 0.75), 0.87 (0.74~ 0.94) and 0.77 (0.73~ 0.80) for male, and 0.73 (0.65~ 0.80), 0.80 (0.71~ 0.86) and 0.82 (0.79~ 0.86) for female, respectively. For overweight/obesity, 11,492 participants were included, out of which 5686 individuals had overweight/obesity. The pooled sensitivity, specificity and AUC (*95%CI*) were 0.83 (0.70~ 0.91), 0.77 (0.66~ 0.85) and 0.86 (0.83~ 0.89) for male, and 0.82 (0.71~ 0.90), 0.84 (0.61~ 0.95) and 0.89 (0.86~ 0.92) for female, respectively. Publication bias was not found after using the Egger’s tests (*P* > 0.05) except in the result of central obesity for females (*P* = 0.013). The efficacy of NC screening obesity and results of Egger’s tests are shown in Table 4. The HSROC curves for male and female are shown in Fig. 3.

**Table 3:** Characteristics of included studies

Author	Year	Country	Age (years)	Gender	Diagnostic criteria	*n*	Cut-off points	TP	FP	FN	TN
Central obesity											
Zhang	2015	China	≥40	Male	WC ≥ 85 cm	3891	36.8 cm	1728	426	604	1133
				Female	WC ≥ 80 cm	5849	32.3 cm	2564	665	1275	1345
Ang	2011	Philippines	49.35 ± 11.26	Male	WC ≥ 90 cm	227	40.0 cm	72	1	44	110
				Female	WC ≥ 80 cm	198	33.8 cm	73	13	35	77
Aswathappa	2014	India	18–65	Male	WC ≥ 90 cm	840	38.0 cm	259	78	99	404
				Female	WC ≥ 80 cm	511	34.0 cm	196	24	127	164
Wang	2017	China	45–86	Male	WC ≥ 85 cm	256	37.0 cm	106	14	35	101
				Female	WC ≥ 80 cm	542	32.7 cm	255	54	85	148
Lin	2017	China	≥50	Male	WC ≥ 90 cm	569	38.5 cm	170	50	56	293
				Female	WC ≥ 85 cm	904	33.4 cm	439	118	79	268
Pei^a^	2017	China	18-25	Male	WC ≥ 85 cm	641	37.1 cm	66	144	20	411
				Female	WC ≥ 80 cm	528	32.6 cm	25	61	5	437
Overweight/obesity											
Kumar	2012	India	≥35	Male	BMI ≥ 25 kg/m^2^	120	38.0 cm	9	17	2	92
				Female	BMI ≥ 25 kg/m^2^	82	34.7 cm	10	4	3	65
Coelho	2016	Brazil	≥60	Male	BMI ≥ 28 kg/m^2^	64	40.5 cm	26	23	4	11
				Female	BMI ≥ 28 kg/m^2^	371	35.7 cm	134	143	26	65
Aswathappa	2014	India	18–65	Male	BMI ≥ 23 kg/m^2^	840	36.0 cm	389	57	157	237
				Female	BMI ≥ 23 kg/m^2^	511	32.0 cm	215	56	121	119
Ben-Noun	2016	Israel	35–65	Male	BMI ≥ 25 kg/m^2^	352	37.0 cm	235	9	2	106
				Female	BMI ≥ 25 kg/m^2^	371	34.0 cm	255	0	3	113
Yan	2014	China	≥65	Male	BMI ≥ 25 kg/m^2^	971	38.0 cm	274	249	41	407
				Female	BMI ≥ 25 kg/m^2^	1121	35.0 cm	322	187	80	532
Yang	2010	China	20–80	Male	BMI ≥ 24 kg/m^2^	1294	38.0 cm	509	122	312	351
				Female	BMI ≥ 24 kg/m^2^	1888	35.0 cm	858	222	389	419
Wang	2017	China	45–86	Male	BMI ≥ 24 kg/m^2^	256	37.2 cm	98	32	28	98
				Female	BMI ≥ 24 kg/m^2^	542	33.2 cm	184	42	66	250
Lin	2017	China	≥50	Male	BMI ≥ 24 kg/m^2^	569	38.4 cm	211	42	69	247
				Female	BMI ≥ 24 kg/m^2^	904	33.7 cm	384	99	93	328
Pei^a^	2017	China	18-25	Male	BMI ≥ 24 kg/m^2^	641	37.4 cm	83	124	34	400
				Female	BMI ≥ 24 kg/m^2^	528	32.2 cm	47	70	13	468

*TP* true positive value, *FP* false positive value, *TN* true negative value, *FN* false negative value

^a^ our unpublished research; *n*: sample size of each study

**Table 4:** The efficacy of neck circumference screening central obesity and overweight/obesity

		N	Sensitivity	Specificity	AUC	*P*
Central obesity						
Male		6424	0.72 (0.68~ 0.75)	0.87 (0.74~ 0.94)	0.77 (0.73~ 0.80)	0.063
Female		8532	0.73 (0.65~ 0.80)	0.80 (0.71~ 0.86)	0.82 (0.79~ 0.86)	0.013
Overweight/obesity						
Male		5107	0.83 (0.70~ 0.91)	0.77 (0.66~ 0.85)	0.86 (0.83~ 0.89)	0.451
Female		6385	0.82 (0.71~ 0.90)	0.84 (0.61~ 0.95)	0.89 (0.86~ 0.92)	0.295

*N* total sample size of each group; *AUC* area under receiver operating curve *P*: *P* values of Egger’s tests

## Discussion

To our knowledge, the meta-analysis on the subject that explored the gender-specific relationship between NC and central obesity as well as overweight/obesity has never been reported. In this study, our own epidemiological research was included to compare the results with that of meta-analysis and improve the representativeness of the study population through acquisition of reliable data from a larger sample size. The results showed that NC may not be a good tool for screening individuals with central obesity. However, it may be a simple and valuable surrogate indicator for BMI, which is significant to identify overweight/obesity in big epidemiological research and in some special occasions or crowd, prevent the development of the overweight/obesity by taking appropriate measures at early stage, and then reduce the incidence or complications of CNCDs.

The prevalence of obesity is increasing at an alarming rate, and the negative implications of this are well known [36]. The WC and BMI are commonly used to identify individuals with central obesity and overweight/obesity. NC reflects the deposits of adipose tissue in the neck, which can be used as an indicator of subcutaneous adipose tissue in the upper-body [37]. The neck is at the junction between the head and the trunk, and is often not covered by clothing, making it easily accessible for measurements. Similarly, NC measurements are less intrusive than those of WC and less cumbersome than those of BMI.

The ROC analysis of this epidemiological study found that NC ≥ 37.1 cm for male and ≥ 32.6 cm for female were determined to be the best cut-off points for screening individuals with central obesity. The thresholds observed by Zhang et al. [27] that studied 9740 participants in Jiangxi, China are similar to this study. Another epidemiological research from Caloocan, Philippines revealed that NC ≥ 40.0 cm for male and ≥ 33.8 cm for female were the best cut-off points for screening individuals with central obesity [28]. In the case of overweight/obesity, NC ≥ 37.4 cm for male and ≥ 32.2 cm for female were determined to be the ideal cut-off points in this study, while the cut-off points reported in India were 36.0 and 32.0 cm for male and female, respectively [29]. The different cut-off points in these populations may have been predetermined by genetic and environmental factors, such as different medical condition and dietary habit. In addition, the objects of our study are undergraduates, so the composition of age is different from other studies, which would cause the inconsistent results among these researches.

Furthermore, we evaluated the efficacy of NC for screening central obesity and overweight/obesity by epidemiological research and meta-analysis. For central obesity, the efficacy of our survey (18–25 years old) was better than that of meta-analysis (≥18 years old); for overweight/obesity, the efficacy of our survey was similar to that of meta-analysis. That is to say, the efficacy of NC as a tool for screening central obesity might be different in each age stage, which may be because age can substantially impact on NC measurements. In this epidemiological study, the efficiency of NC for screening individuals with both central obesity and overweight/obesity in female was higher than in male. ROC curves showed that the best threshold of NC for screening individuals with central obesity and overweight/obesity might be different between male and female. Meta-analysis showed that NC predicted overweight/obesity better than central obesity, which suggested that NC may be a simple and valuable surrogate indicator for BMI, especially in female group. Reasons for the different efficacy between genders and types of obesity might be due to differences in body composition, sex hormone, distribution of adipose tissue and activity intensity between male and female. Studies have suggested that sex hormones may regulate body fat distribution [38]. Androgen plays a key role in visceral adipose tissue accumulation in abdominal, while, estrogen can promote abdominal visceral adipose tissue transfer to subcutaneous and peripheral region [39]. The main adipose in the neck is the subcutaneous adipose tissue. Studies have indicated that the correlation coefficient of subcutaneous adipose tissue and BMI was larger than that of subcutaneous adipose tissue and WC [40, 41]. Therefore, NC could be a better indicator for screening overweight/obesity, especially in female, which was consistent with most of included publications. Furthermore, the results of meta-analysis revealed that there was substantial heterogeneity between studies and the HSROC curves were asymmetric except the curve of overweight/obesity for female. There might be several reasons that can explain these phenomena. (1) The number of satisfactory studies for meta-analysis was limited due to the less research for NC screening central obesity and overweight/obesity. (2) The variations in sample sizes and participants’ characteristics of each study may introduce heterogeneity between studies. (3) The critical value of WC and BMI for determining central obesity and overweight/obesity was diverse in different studies, which may affect the HSROC curves of meta-analysis.

In aggregate, the results of the present epidemiological research and meta-analysis suggested that NC may not be a good tool for screening individuals with central obesity. However, it may be a valuable tool for screening individuals with overweight/obesity, especially in female. From a public health perspective, it is valuable to be able use NC to assess overweight/obesity because it saves time and allows clinicians and researchers to increase the number of subjects investigated, especially in some special occasions, such as for expectant mothers, athletes and patients with ascites. Thus, NC might be a better surrogate index for screening overweight/obesity. Compared to WC and BMI, there are several unique advantages for NC. The measuring tool of NC is simple and can be carried conveniently. In winter, the use of thick clothing may erroneously give larger than actual WC and BMI values. The NC can be measured easily without considering the thickness of an individual’s clothes [34, 42]. Additionally, NC cannot be affected by factors like meal, respiration or health conditions. For expectant mothers, NC could evaluate the levels of obesity better than WC and BMI, which can prevent the development of gestational diabetes mellitus and pregnancy-induced hypertension syndrome by taking appropriate measures when a lager NC was observed. Besides, NC is associated with MS, obstructive sleep apnea and cardiometabolic risk factors [14]. Study showed that the relationship between MS and NC was stronger than that with WC [30]. Therefore, identification of obesity in early stage, controlling weight and improving lifestyle on time will certainly permit drastic reductions in risk of MS and other CNCDs. Unfortunately, measurement of NC is not suitable for patients with certain diseases, such as goiter or neck tumor, otherwise it may overrate the prevalence of obesity. However, this study has some limitations. First, the number of studies included in the meta-analysis was small. In addition, all of the included participants were from Asia except the study by Coelho et al. Therefore, subgroup analysis based on age groups and continents were not conducted, and the cut-off values of NC we got cannot be generalized to a larger population. Second, the critical values of WC and BMI for determining central obesity and overweight/obesity were different in some studies, which resulted in the inevitable heterogeneity in the meta-analysis. Third, there was potential publication bias in the studies that used NC screening for central obesity in females, possibly due to different characteristics and limited number of included researches. Therefore, it would be helpful to examine these findings in other ethnic groups using larger samples, and increase the number of studies for meta-analysis, while subgroup analyses as well as meta-regression could be performed for age, region, and cut-off values of central obesity and overweight/obesity in future studies.

## Conclusions

There are moderate correlations between NC and obesity indicators like WC and BMI. NC may not be a good tool for screening individuals with central obesity. However, it may be a simple and valuable surrogate indicator for BMI, especially in females.

## Abbreviations

+LR: positive likelihood ratio
AUC: area under receiver operating curve
BFP: body fat percentage
BMI: body mass index
CI: confidence interval
CNCD: chronic non-communicable disease
CNKI: China National Knowledge Infrastructure
CVD: cardiovascular disease
DM: diabetes mellitus
DOR: diagnostic odds ratio
FN: false negative value
FP: false positive value
HC: hip circumference
HSROC: hierarchical summary receiver operator characteristic
MS: metabolic syndrome
NC: neck circumference
PRISMA: Preferred Reporting Items for Systematic reviews and Meta-Analyses
ROC: receiver operator characteristic
TN: true negative value
TP: true positive value
WC: waist circumference
WHR: waist-to-hip ratio
